# Persistent detection of Zika virus RNA from an infant with severe microcephaly – a case report

**DOI:** 10.1186/s12879-018-3313-4

**Published:** 2018-08-10

**Authors:** Carlos A. A. Brito, Adélia Henriques-Souza, Cynthia R. P. Soares, Priscila M. S. Castanha, Laís C. Machado, Mylena R. Pereira, Mariana C. M. Sobral, Antonio R. Lucena-Araujo, Gabriel L. Wallau, Rafael F. O. Franca

**Affiliations:** 1 Department of Clinical Medicine, Federal University of Pernambuco – UFPE, Recife, 50670-901 Brazil; 2 Department of Pediatrics, Hospital da Restauração, Recife, 50110-900 Brazil; 3 Department of Virology and Experimental Therapy, Aggeu Magalhães Institute, Oswaldo Cruz Foundation – FIOCRUZ, Avenida Professor Moraes Rego s/n, Recife, PE 50740-465 Brazil; 4 Department of Entomology, Oswaldo Cruz Foundation - FIOCRUZ, Aggeu Magalhães Institute, Recife, 50740-465 Brazil; 5 Department Veterinary Medicine, Federal Rural University of Pernambuco – UFRPE, Recife, 52171-900 Brazil; 6 Department of Biophysics, Federal University of Pernambuco – UFPE, Recife, 50670-901 Brazil

**Keywords:** Zika virus, Neurological disease, Microcephaly, Virus persistence

## Abstract

**Background:**

Zika virus (ZIKV) is a recently emerged arbovirus, which infection during pregnancy is associated with a series of congenital malformations, collectively denominated Congenital Zika Syndrome (CZS). Following infection, ZIKV RNA has a median duration period of 10 days in plasma and up to 6 months in semen in immunocompetent adult individuals. Moreover, ZIKV is able to replicate and persist in fetal brains and placentas, consequently, infection is associated with pregnancy loss, albeit the pathogenic mechanisms are still unknown.

**Case presentation:**

Here we report a CZS case of an infant born during the ZIKV outbreak in northeast Brazil, the child presented recurrent episodes of seizures with prolonged presence of ZIKV RNA on the central nervous system (CNS) and blood. ZIKV RNA was identified and partially sequenced from a sample of cerebrospinal fluid (CSF) obtained from the infant with 6 months of life, and later from another sample after the infant completed 17 months of life. Commonly congenital infections were discarded based on STORCH (syphilis, toxoplasmosis, rubella, cytomegalovirus and herpes simplex virus) negative laboratory results. Presence of specific ZIKV antibodies on both mother and children confirmed the association of severe microcephaly and ZIKV infection, diagnosed after birth.

**Conclusions:**

Altogether, our data raise the possibility that CZS cases may result in prolonged viral presence, these findings could be useful for therapy and diagnostic recommendations.

## Background

Zika virus (ZIKV) was firstly isolated from rhesus macaques in the Zika forest of Uganda in 1947 [1]. The virus is mainly transmitted by infected *Aedes* mosquitoes, however other modes of infection have been reported, such as sexual and perinatal transmission [2]. Infections are usually not detected (asymptomatic), although 20% of the infected individuals progress to a clinically apparent febrile illness with commonly reported symptoms including mild fever, rash, arthralgia, headache, and conjunctivitis [3]. Additionally, severe neurologic manifestations such as Guillain-Barre Syndrome (GBS) in adults and the newly described Congenital Zika Syndrome (CZS), a wide spectrum of congenital malformations in fetuses and newborns, have been described [4]. ZIKV attracted a lot of attention after a large outbreak in Brazil in 2015, which was associated with an increased number of microcephaly cases (a congenital malformation where the head circumference is smaller than 2 standard deviations below the mean for the same age and sex) [5].

ZIKV immunopathogenesis is not completely understood. As reported for other viruses of the same family, ZIKV infection is usually self-limited, resulting in viral clearance in approximately 1 week after infection, although prolonged viremia has been documented, especially in pregnant women and in the semen of infected men [6-9]. ZIKV is highly neurotropic and replicates in the central nervous system (CNS). In fact, in situ hybridization screening demonstrated the presence of ZIKV RNA in brains of fetuses from pregnancy losses [10] and different experimental approaches identified ZIKV in the CNS of infected animals at different times post-infection [11, 12]. Interestingly, a recent study demonstrated that in blood, ZIKV is rapidly controlled, however, the virus is able to persist for long periods in the CNS (up to 42 days in cerebrospinal fluid (CSF) of experimentally infected Rhesus monkeys) [13].

ZIKV infection can be diagnosed through viral RNA detection by the use of Real-Time Reverse-Transcriptase–Polymerase-Chain-Reaction (rRT-PCR) assays. The presence of anti-ZIKV IgM antibodies is also an indication of recent infection, although the complete kinetics of IgM production have not yet fully described [14]. Moreover, the presence of anti-Zika IgM antibodies in the CSF of microcephaly-diagnosed neonates is a confirmatory test in cases where Zika infection during pregnancy is suspected [15]. Experimentally, ZIKV is able to infect human neural progenitor cells (NPC), impairing their development [16]. In mice, ZIKV infection results in cell-cycle arrest, apoptosis, and inhibition of NPC differentiation, which results in cortical thinning and microcephaly [17, 18]. Altogether, these findings confirm that ZIKV is able to directly infect the CNS and several independent publications have demonstrated the role of ZIKV in microcephaly development [19]. However, the clinical evolution of the infants diagnosed with microcephaly, due to the direct infection of the CNS during pregnancy, has not yet been assessed.

## Case presentation

**Fig. 1:**
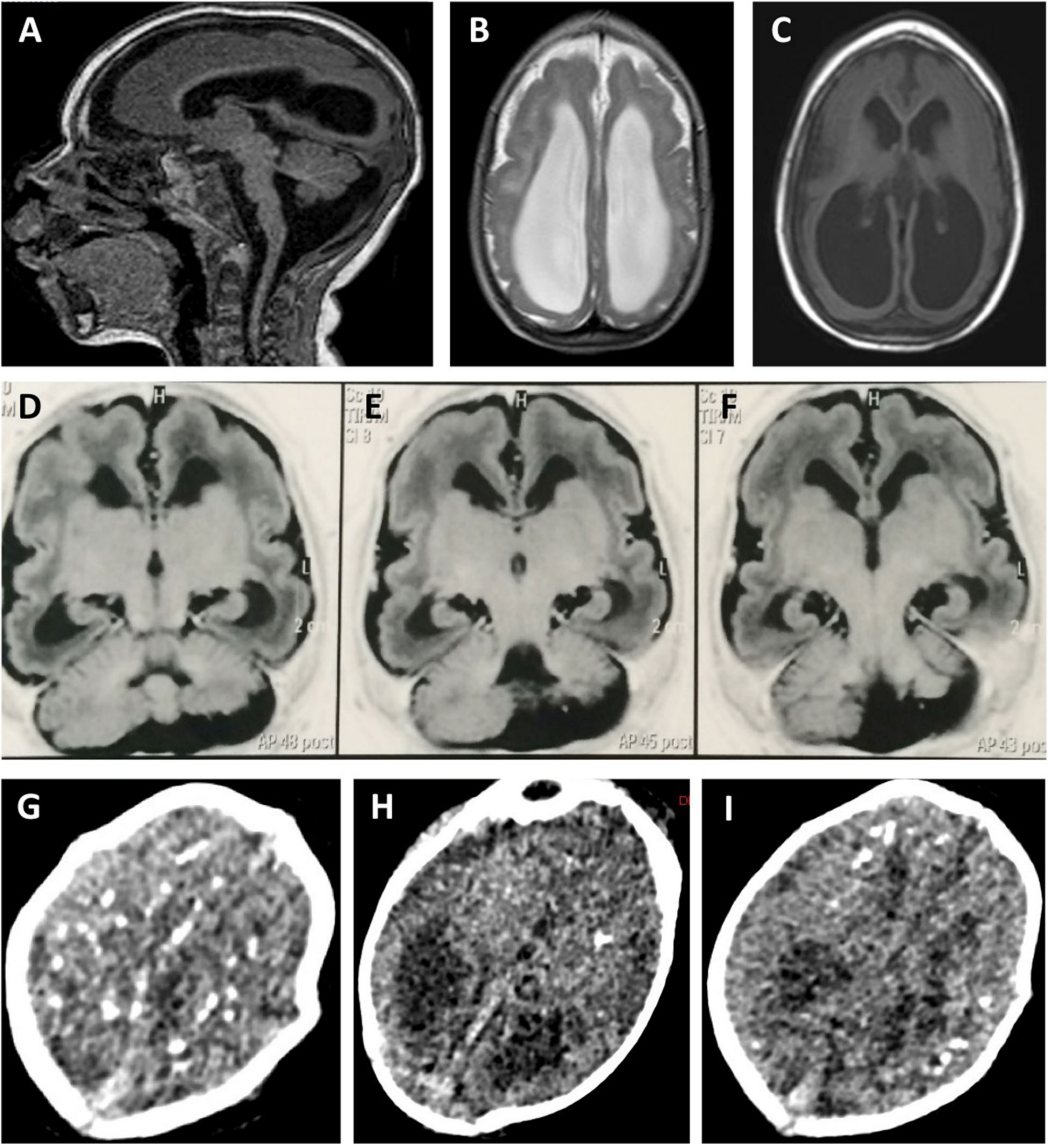
Brain Imaging after birth. **a** MRI Sagittal T1 weighted image: hypogenesis of the corpus callosum, enlarged cisterna magna, and ventriculomegaly. **b** MRI Axial T2 weighted image: simplified frontal gyral pattern, ventriculomegaly. **c** Axial T1 weighted image: pachygyria/lissencephaly in the frontal lobe, ventriculomegaly. **d**, **e**, **f** MRI Axial T1 weighted image: simplified frontal gyral pattern, ventriculomegaly, cerebellum hemisphere hypoplasia. **g**, **h**, **i** Axial non-contrast CT image: multiple bilateral calcifications in the junction between cortical and subcortical white matter, ventricular enlargement

**Fig. 2:**
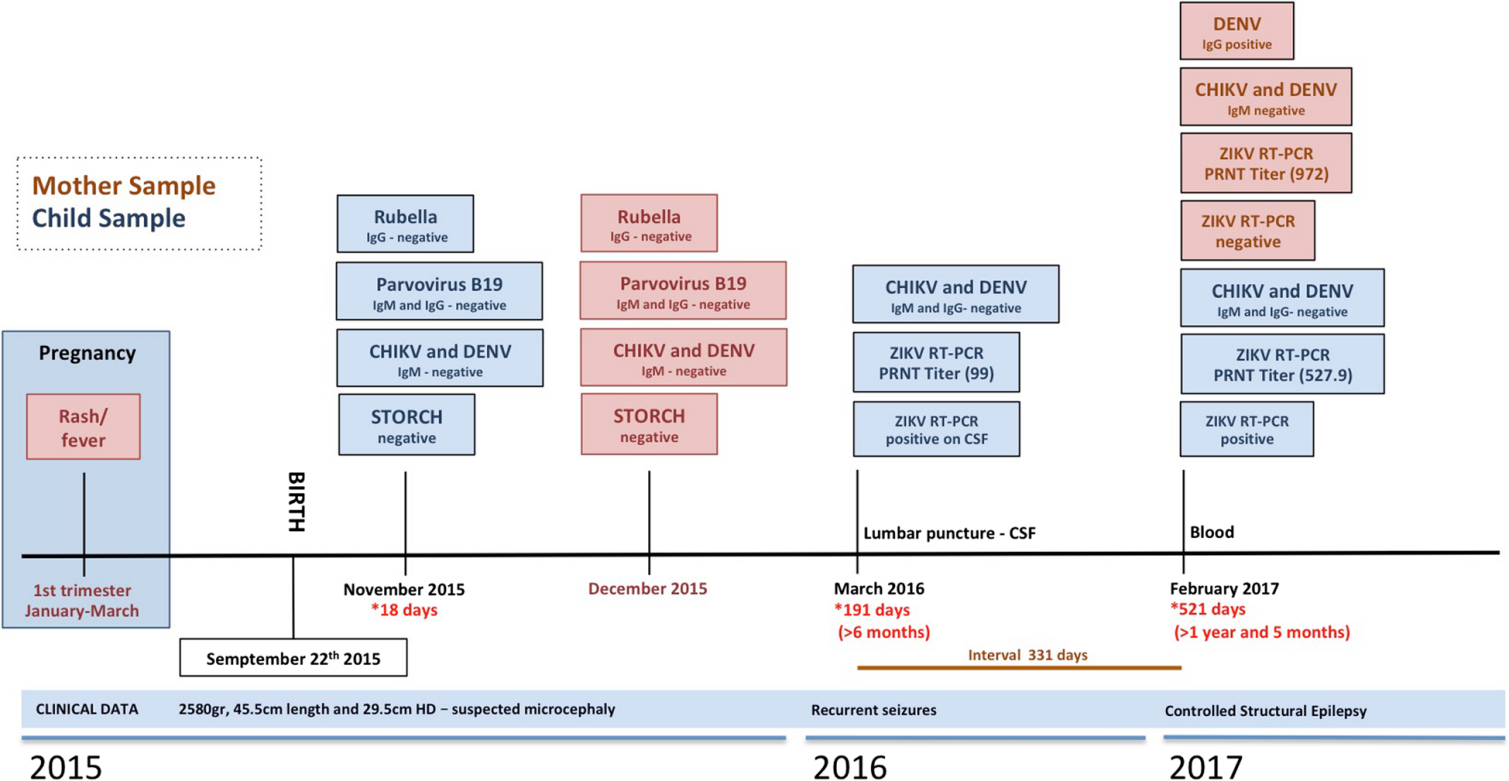
Clinical and laboratorial timeline of a persistent Zika virus Severe Microcephaly case. The panel shows the clinical and laboratory results of pregnancy and birth, mother and child samples (as designated on figure) were tested accordingly to Brazilian Ministry of Health protocol for Congenital Zika Syndrome investigation, as described in material and methods. Principal clinical findings are shown in the bottom. STORCH denotes a group of laboratory tests comprising syphilis, toxoplasmosis, rubella, cytomegalovirus infection, and herpes simplex viruses; maximum interval denotes the results from two consecutive samples with positive laboratory result for ZIKV on rRT-PCR protocol. PRNT plaque reduction neutralization test

In September 2015, a middle age woman (44 years old) resident from Recife, northeast Brazil, gave birth to a male in a local public hospital. At birth, the neonate had 2580 g, 45.5 cm in length and head circumference of 29.5 cm (suspected case of microcephaly). The child was born after a 38 weeks single-gestation period (full-term), during the first trimester of pregnancy the mother reported a febrile episode followed by headache, joint pain, and rash, the symptoms described did not last more than 3 days and no other symptoms were reported, dengue virus (DENV) IgM serology was negative. At 20 weeks of the gestational age, a prenatal intrauterine ultrasound was performed and the diagnosis was consistent with congenital microcephaly. After birth, during the first month of life, a complete brain imaging examination of the infant evidenced the findings consistent with severe microcephaly, following the protocol of the Brazilian Ministry of Health for Microcephaly Investigation. Brain imaging (magnetic resonance) demonstrated the presence of lissencephaly, decreased brain parenchymal volume, decreased cortical mantle and white matter together with hypoplasia of the corpus callosum. Computed tomography and transfontanellar cranial ultrasound evidenced the presence of multiple brain calcifications, colpocephaly and gliosis in the left cerebellar hemisphere were also documented (Fig. 1a-i). Serological tests were performed on the mother (18 days after she gave birth), the results for STORCH laboratory screen (syphilis, toxoplasmosis, rubella, cytomegalovirus and herpes simplex virus), Parvovirus B19 IgM and chikungunya virus (CHIKV) IgM were all negative (Fig. 2), no ZIKV serological (ELISA or PRNT_50_) or molecular tests (rRT-PCR) were performed during pregnancy since these tests were not available at that time, being implemented in Brazil only later in 2016.

Laboratory STORCH tests were also performed in the child with 1 month of life, the results were all negative, including chikungunya IgM and anti-Epstein-Barr virus (EBV) IgM (Fig. 2). No Zika virus serology or molecular tests were performed on the child and no samples were stored for later investigations. After 1 month of life, a detailed neurological examination in the infant evidenced upper and lower limb spasticity (mainly on upper limbs), extreme irritability, continuous cry and head circumference measurement of 31 cm was consistent with severe microcephaly (< 3 SD on the Fenton growth chart).

With approximately 6 months of life, the child was admitted to a local hospital after presenting several recurrent seizure episodes. Electroencephalographic (EEG) examination evidenced the presence of focal frontal epileptiform discharges. At the hospital, a blood and a cerebrospinal fluid (CSF) samples were collected. Laboratory results of serum and CSF confirmed the presence of ZIKV viral RNA on both samples, analyzed by rRT-PCR following a well-established laboratory protocol [20]. Additionally, rRT-PCR for chikungunya (CHIKV) and ELISA tests were all negative in serum and CSF as follows anti-ZIKV IgM, anti-DENV I, M and IgG, anti-CHIKV IgM and IgG (Fig. 2). To confirm ZIKV infection a plaque reduction neutralization test (PRNT_50_) was performed from the CSF sample, a ZIKV positive neutralization titer of 99 confirmed the previous infection, no neutralizing DENV antibody titers were observed (Table 1 and Fig. 2).

**Table 1:** Plaque Reduction Neutralization Test (PRNT) for Zika virus (ZIKV) and dengue virus (DENV1–4) in maternal serum, child serum and cerebrospinal fluid (CSF) specimens collected at different ages of the neonate with Congenital Zika Syndrome

Sample	Age at testing	PRNT_50_ Titer				
		ZIKV	DENV-1	DENV-2	DENV-3	DENV-4
Mother (serum)	17 months^a^	972	< 20	< 20	689.5	262.7
Neonate						
Serum	17 months	527.9	< 20	< 20	< 20	< 20
CSF	6 months	99	< 20	< 20	< 20	< 20

^a^Age of the child at the time of sample collection

The interesting presence of ZIKV RNA previously identified at 6 months of life prompted us to investigate ZIKV persistence in a second sample collected with the age of 1 year and 5 months (here denominated 17 months for a better understanding). Given the risk of an invasive lumbar puncture, only a blood sample was collected from the child following a regular visit to the pediatrician, on the same occasion, a blood sample from the mother was also requested. On child serum, after 17 months of life, ZIKV rRT-PCR was still positive. Serology was negative for the tested arboviruses as follows anti-ZIKV IgM, anti-DENV IgM and IgG, anti-CHIKV IgM and IgG (Fig. 2). A plaque reduction neutralization test (PRNT_50_) was again performed on child serum, ZIKV positive neutralization dilution titer of 527.9 reinforced our previous result of ZIKV infection, and again no neutralizing DENV antibody titers were observed. Moreover, the presence of a high titer of ZIKV neutralization antibodies (titer of 972) on mother’s serum confirms Zika virus infection (Table 1 and Fig. 2). On mother’s serum the presence of positive DENV neutralization titer is not surprisingly since dengue virus is endemic in Brazil, especially in the northeast region.

**Fig. 3:**
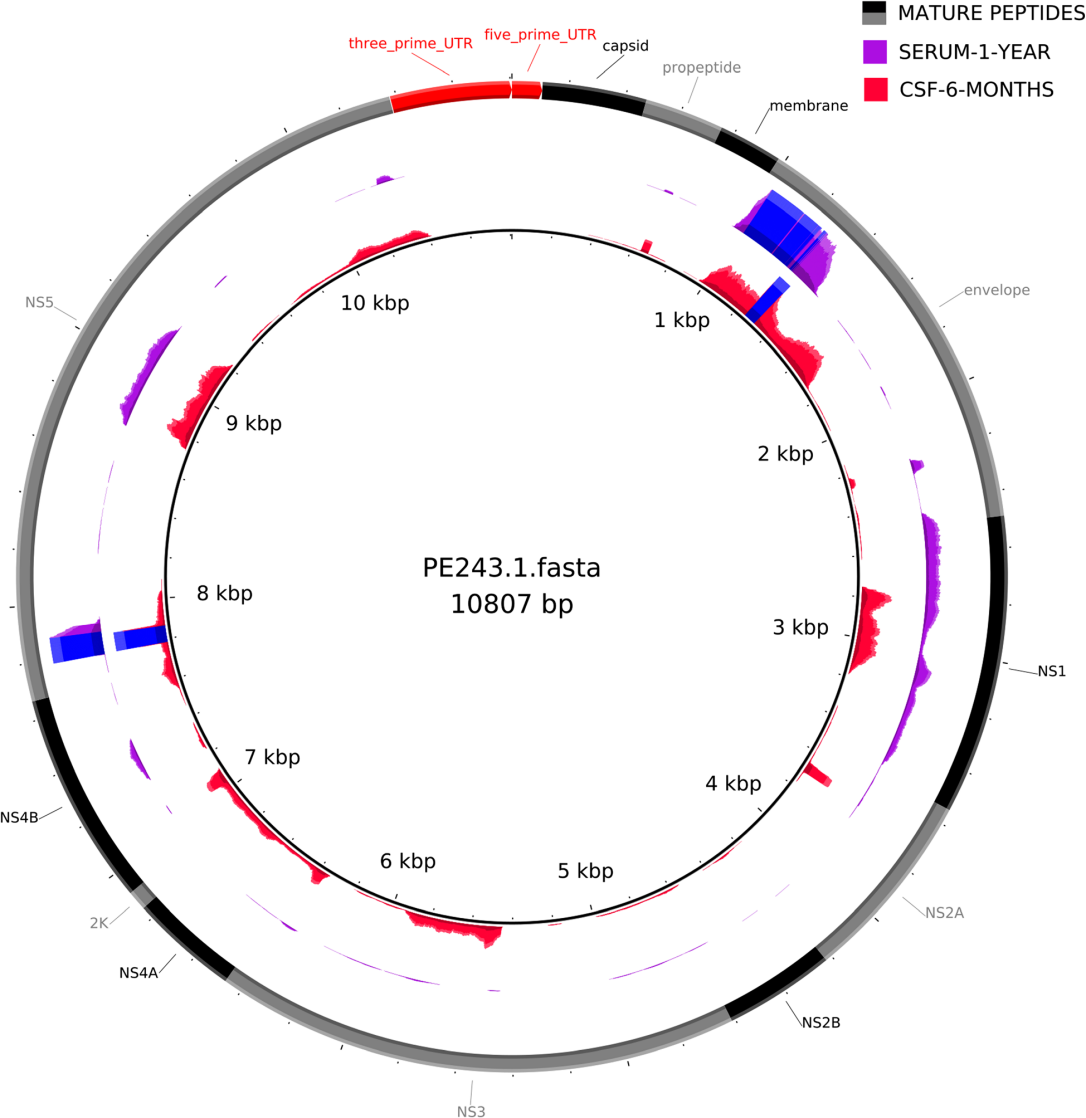
ZIKV partial genomes obtained from samples. Read mapping pattern on the PE243 reference genome. Blue rectangles show coverage depth higher than 100 reads. The most external ring is the annotation of mature peptides (black and grey) and untranslated regions (red) of the ZIKV genome

**Fig. 4:**
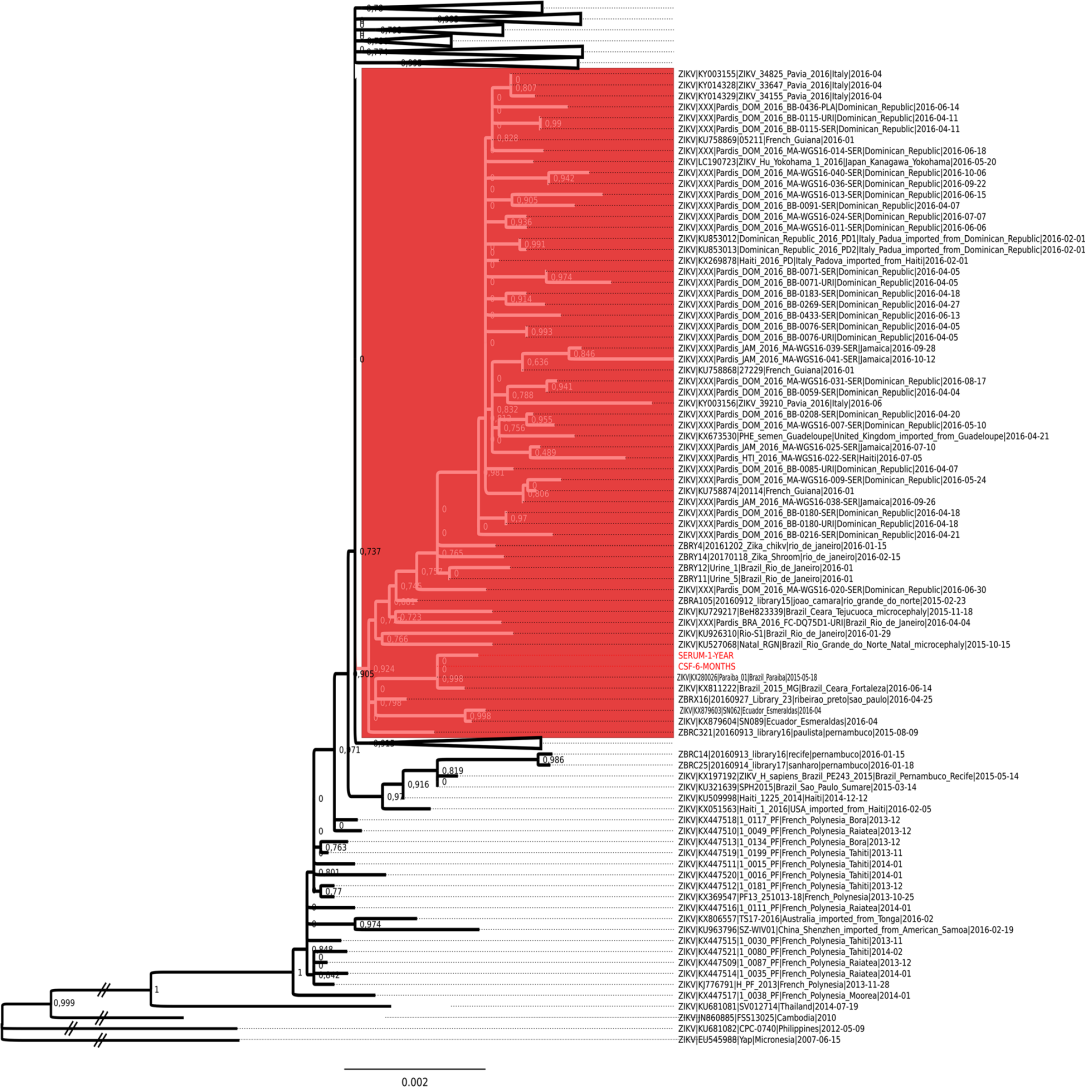
ZIKV whole-genome phylogenetic analysis of the Asian lineage by maximum likelihood. The red shaded clade is on of the large polytomic tree of epidemic ZIKV where draft genomes obtained in this study clustered (red tree tips). Other large clusters were collapsed for clarity. Numbers close to nodes are the SH-like node support

It’s intriguing the presence of ZIKV RNA for such a long time and since samples were stored with RNA preservative solution viral isolation was not attempted. Thus, to further confirm the presence of ZIKV on child CSF and serum we performed genome sequencing direct from blood and CSF positive samples, employing a previously published protocol [21]. We obtained two partial ZIKV genomes from samples CSF-six-months and serum-17-months of 6653 and 5292 base pairs, respectively, corresponding to 60.6 and 48.9% of genomic coverage (Fig. 3). Phylogenetic analysis showed that these two draft genomes belong to the Asian genotype and clustered closely with a ZIKV isolate from Paraíba/Brazil (Fig. 4), located around 120 km from where the mother gave birth. Since six-months-CSF sample differs from Paraiba_01 ZIKV strain by two single nucleotide polymorphisms (SNPs) in the envelope gene (E) and serum-17-months sample differs from Paraíba_01 by three SNPs, these results are consistent with a single ZIKV infecting strain. The child is being followed in a local hospital. Clinical evolution consists mainly in delayed neuropsychomotor development, dysphagia (difficulty swallowing), visual impairment, and double spastic hemiplegia. Treatment consists of clobazam (antiepileptic drug). After almost 2 years of life, the child presents controlled structural epilepsy.

### Sample collection and processing

CSF and blood were collected through standard procedures. Anti-dengue and anti-chikungunya virus IgM and IgG antibodies were detected by commercially available capture ELISA kits (Anti-Dengue Virus ELISA IgM/IgG and Anti-CHIKV IgM/IgG from EuroImmun AG, Luebeck - Germany), following manufacturers instructions. Serotype-specific anti-DENV and anti-Zika antibodies were assessed by plaque reduction neutralization test (PRNT). The antibody titer was determined as the serum dilution that inhibited 50% of the tested virus inoculum (PRNT_50_). Anti-Zika IgM antibodies were detected by Capture Enzyme-Linked Immunosorbent Assay (MAC-ELISA). Viral RNA was extracted by the use of a QIAamp Viral RNA kit (Qiagen, Hilden - Germany), following manufacturers instructions and rRT-qPCR reactions were performed from purified RNA serum samples accordingly to Lanciotti et al.[20], with modifications. Briefly, reactions were performed with the kit GoTaq® Probe 1-Step RT-qPCR System (Promega, Fitchburg, USA) in a 20 μl final volume, primers and probes employed were as follows: **Zika 1087** 5’-CCGCTGCCCAACACAAG-3′, **Zika1163c** 5’-CCACTAACGTTCTTTTGCAGACAT-3′ and **Zika 1087 VIC (probe)** 5’-AGCCTACCTTGACAAGCAGTCAGACACTCAA-3′. Samples with a Ct value < 38 in duplicate wells were considered to be positive for ZIKV. For sequencing, total RNA extracted as above was converted to cDNA and amplified, PCR products were then quantified and libraries were prepared with Nextera XT Library Prep Kit (Illumina, San Diego - USA), MiSeq Reagent Kit V3 of 150 cycles was used to sequence employing a paired-end strategy. Mapped reads were visualized and majority rule consensus genomes were extracted with Integrated Genome Viewer (IGV) and carefully checked on the mapping results. Phylogenetic reconstruction was performed on an alignment of the coding region of all ZIKV genomes available by maximum likelihood. All sequences were aligned with Mafft v 7.

## Discussion

Here, we reported a case of an infant diagnosed with severe microcephaly presenting virus persistence for several months after birth. The child was born in 2015, during the ZIKV outbreak in Brazil, the symptoms reported by the mother during pregnancy are consistent with ZIKV infection, albeit laboratory confirmation has not been performed since ZIKV specific detection protocols were only implemented later in Brazil. On the other hand, after giving birth, a complete laboratory screening for the most common congenital infections was performed and the results were all negative. At birth, the neonate was not tested for ZIKV infection, however, the presence of ZIKV-neutralizing antibodies on a CSF sample collected 6 months after birth confirms the previous infection. Based on that, a strong argument between the clinical signs of severe microcephaly and ZIKV infection can be implied. In fact, detection of anti-ZIKV IgM antibodies in the CSF is strongly associated with ZIKV congenital infection [15]. Moreover, following infection, ZIKV IgM antibodies increases from 4 to 7 days, persisting for several weeks. On the other hand, neutralizing antibodies are long-lasting. Here the presence of ZIKV neutralizing antibodies is a confirmation of ZIKV exposure, albeit the participation of maternally transferred antibodies should be also considered. During the clinical interview we did not include data about breastfeeding, and since dysphagia, including breastfeeding difficulties, has been documented in several children with CZS [22], we could not correctly evaluate the role of maternally transferred antibodies in the child immune response.

After recurrent seizure episodes followed by the need for hospitalization, the neurologist performed a CSF sampling from the child with the age of 6 months and laboratory RT-PCR analysis identified the presence of ZIKV on CSF. Since birth, the child has been followed by a team of pediatricians for treatment and early stimulation, thus after 17 months of life, the child had another sample collected which was tested positive for ZIKV presence. The maximum interval between consecutive ZIKV rRT-PCR positive samples from this case was of 331 days, to our knowledge, this is the longest report of ZIKV persistence in a single individual. Virus persistence for such a long time has several implications: i) infected individuals could act as reservoirs contributing to maintenance of virus circulation; ii) continuous immune stimulation, by the presence of ZIKV in different body tissues, can result in recurrent organ damage with consequent disease worsening; iii) virus persistence on CZS cases may require differential treatment (e.g., use of antivirals associated with anti-inflammatory drugs). By comparing the levels of ZIKV-neutralizing antibodies from early samples, we could have captured a better picture of the child’s immunity, unfortunately, we were not able to access these samples (first month of life). These findings presented here are certainly not common to all CZS cases and could be partially explained by i) re-infection – the infant was born during a highly ZIKV circulation period and since protection of neonates are mostly acquired by maternal antibodies transference on breast milk and especially by the fact the adaptive immune response on early life is still on development (characterized by higher induction of Th2-cell polarizing cytokines and suboptimal Th1 responses and B-cell differentiation), neonates are more susceptible to different pathogens [23], thus, the child may have been re-infected after birth; ii) infection during fetal development may lead to tolerization – in this scenario T and B cell responses are not effective, consequently contributing to enhanced infection susceptibility.

The ZIKV microcephaly outbreak was first documented in Brazil in 2015, and although neuro-developmental malformations have been linked to many other different viral infections, ZIKV pathogenesis is still not fully understood. However, it is well accepted that infection during the first trimester of pregnancy may result in congenital malformations [21]. Zika viral RNA was found on saliva [24], amniotic fluid [25], urine [26], cerebrospinal fluid (CSF), blood, semen and tears [27, 28]. Interestingly, ZIKV can persist on different body compartments for longer periods. In fact, experimentally infected rhesus macaques presented ZIKV viral RNA for several weeks post infection in neuronal, lymphoid, joint/muscle and male/female reproductive tissues [13]. Additionally, in cases of human infection, the virus can persist for more than 6 months on semen [7].

## Conclusion

Here, we added more data about ZIKV persistence, even if it is based on a single case. Clearly, we cannot rule out the possibility of two independent Zika infections between the different time points analyzed, albeit both samples were sequenced and results are consistent with a single infecting strain. Our results here presented shows compelling evidence of chronification of infection, which has not yet been investigated in CZS cases. Based on that, we propose new studies to better understand the clinical evolution of the CZS documented cases.

## Abbreviations

cDNA: Complementary deoxyribonucleic acid
CHIKV: Chikungunya virus
CNS: Central nervous system
CSF: Cerebrospinal fluid
CZS: Congenital Zika Syndrome
DENV: Dengue virus
EBV: Epstein-Barr virus
EEG: Electroencephalography
ELISA: Enzyme-Linked Immunosorbent Assay
GBS: Guillain-Barre Syndrome
IgG: Immunoglobulin G
IgM: Immunoglobulin M
NPC: Neuroprogenitor cells
NS5: Nonstructural protein 5
PCR: Polymerase chain reaction
PRNT_50_: Plaque Reduction Neutralization Test 50%
RNA: Ribonucleic acid
rRT-PCR: Real Time Reverse-Transcriptase–Polymerase-Chain-Reaction
SNPs: Single nucleotide polymorphisms
STORCH: A list of laboratory tests encompassing Syphilis, Toxoplasma gondii, Rubella, Cytomegalovirus and Herpes Simplex virus
ZIKV: Zika virus
